# Thromboembolic Events in the Era of COVID-19: A Detailed Narrative Review

**DOI:** 10.1155/cjid/3804576

**Published:** 2025-03-04

**Authors:** Maria Abou Mansour, Christophe El Rassi, Bshara Sleem, Raphah Borghol, Mariam Arabi

**Affiliations:** ^1^Faculty of Medicine, American University of Beirut Medical Center, Beirut, Lebanon; ^2^Pediatric Department, Division of Pediatric Hematology-Oncology, American University of Beirut Medical Center, Beirut, Lebanon; ^3^Pediatric Department, Division of Pediatric Cardiology, American University of Beirut Medical Center, Beirut, Lebanon

## Abstract

COVID-19, caused by the SARS-CoV-2 virus, is not only characterized by respiratory symptoms but is also associated with a wide range of systemic complications, including significant hematologic abnormalities. This is a comprehensive review of the current literature, using PubMed and Google Scholar, on the pathophysiology and incidence of thromboembolic events in COVID-19 patients and thromboprophylaxis. COVID-19 infection induces a prothrombotic state in patients through the dysregulation of the renin–angiotensin–aldosterone system (RAAS), endothelial dysfunction, elevated von Willebrand factor (vWF), and a dysregulated immune response involving the complement system and neutrophil extracellular traps (NETs). As a result, thromboembolic complications have emerged in COVID-19 cases, occurring more frequently in severe cases and hospitalized patients. These thrombotic events affect both venous and arterial circulation, with increased incidences of deep venous thrombosis (DVT), pulmonary embolism (PE), systemic arterial thrombosis, and myocardial infarction (MI). While DVT and PE are more common, the literature highlights the potential lethal consequences of arterial thromboembolism (ATE). This review also briefly examines the ongoing discussions regarding the use of anticoagulants for the prevention of thrombotic events in COVID-19 patients. While theoretically promising, current studies have yielded varied outcomes: Some suggest potential benefits, whereas others report an increased risk of bleeding events among hospitalized patients. Therefore, further large-scale studies are needed to assess the efficacy and safety of anticoagulants for thromboprophylaxis in COVID-19 patients.

## 1. Introduction

In December 2019, emerging cases of respiratory infections with unknown etiology were reported in Wuhan, China [1, 2]. The culprit of this new disease termed severe acute respiratory syndrome (SARS) was found to be the SARS-CoV-2 virus, a new member of the Corona family of viruses [1, 3]. The virus rapidly spread worldwide and became a true global health threat due to its high virulence and its ability not only to cause respiratory symptoms, but also to involve other organ systems such as the neurologic, gastrointestinal, cardiac, and, to our interest, hematologic system [1, 4]. Furthermore, according to recent reports, SARS-CoV-2 has been the cause of almost 700 million positive cases of COVID-19 and over 7 million deaths [5, 6].

During viral or bacterial infections, the coagulation cascade gets activated as a host defense mechanism to limit the spread of the pathogen [7]. However, hyperactivation of the coagulation cascade can lead to clot formation [8].

Clot formation or hemostasis is a physiological process that results in the formation of a platelet plug or thrombus in the case of vascular damage [9]. In 1856, the German physician Rudolf Virchow established a triad defining the predisposing factors of thrombosis: alterations in normal blood flow, endothelial cell injury, and/or a hypercoagulable state [10, 11]. In brief, when injury to a vessel wall occurs, circulating platelets adhere, aggregate, and finally cover the damage to prevent blood leakage [9, 10]. Another essential aspect of hemostasis is clot dissolution or fibrinolysis, which, if dysregulated, could lead to the maintenance of the clot formed, also known as thrombosis [10]. In fact, according to Andrews and Berndt, both physiological and pathological blood clot formation share the same pathway, but while the former is protective, the latter leads to thromboembolic diseases [9]. They comprise deep venous thrombosis (DVT), pulmonary embolism (PE), and other associated cardiovascular conditions such as ischemic stroke (IS) and myocardial infarction (MI) [12–14].

Since the beginning of the COVID-19 pandemic, such thromboembolic events have been on the rise in patients infected by the virus [13–16]. Whether they are part of the pathogenesis in this respiratory disease or a complication of its actions at the cellular level [14, 15] is an essential question we will base our discussion on. In effect, an increasing number of COVID-19 patients, especially those with severe illness, showed hematological events such as venous and arterial thromboses, with presentations such as DVT, IS, thrombosis-related MI, and systemic arterial embolism [13, 16]. This article aims to discuss the pathophysiology of thromboembolic events during COVID-19 and following vaccination against SARS-CoV-2, their relative incidence, severity, and treatment options.

## 2. Methods

This literature review was performed in June 2024. The review was conducted primarily via PubMed, EMBASE, and Google Scholar, using the keywords: thromboembolic events—thrombosis—DVT—PE—bleeding—pathophysiology—immune response—thromboprophylaxis, combined with COVID-19—COVID-19 vaccination—vaccine-induced immune thrombotic thrombocytopenia (VITT) as MeSH terms for our search. Articles examining the link between COVID-19 and thromboembolic events were gathered, and their findings were summarized in our review. The retrieved articles were verified for accuracy, relevance, and validity. Accuracy was confirmed by cross-referencing findings with other sources and validity by appraisal of studies according to established critical appraisal tools relevant to their respective designs. For instance, observational studies included were evaluated using the Newcastle–Ottawa scale and only were included studies with a score of at least 6 (over 9). Relevance was then assessed by matching the studies' findings with our review objectives. No limitations on the type of studies or country of origin were made. Articles were referenced only if written or translated in English regardless of the language of origin. The search was done within the 2020–2024 period. Other papers were considered from the references of the articles chosen.

## 3. Results

### 3.1. Pathophysiology of COVID-19-Induced Thrombosis

Different mechanisms are involved in the prothrombotic state of COVID-19 patients, including a dysregulated renin–angiotensin–aldosterone system (RAAS), endothelial dysfunction, elevated von Willebrand factor (vWF), and a dysregulated immune response.

**Figure 1:**
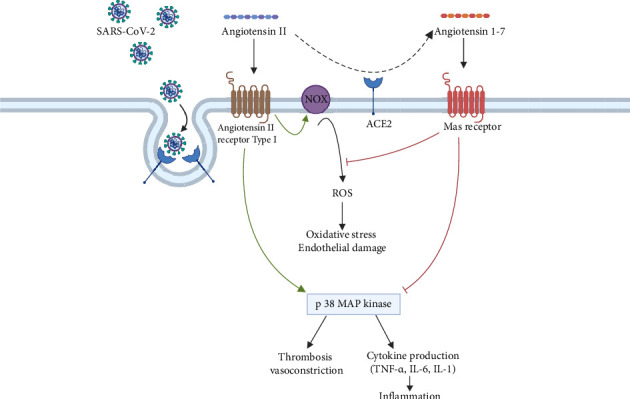
Dysregulation of the renin–angiotensin–aldosterone system following SARS-CoV-2 infection.

The coronavirus SARS-CoV-2 has been shown to dysregulate the RAAS, initiating a cascade of events that exacerbates coagulopathy and contributes to the severe clinical manifestations observed in COVID-19 patients [17]. First, the spike protein of Coronavirus mediates the virus's entry into target cells [18]. The spike subunits of both SARS-CoV and SARS-CoV-2 interact with the angiotensin-converting enzyme 2 (ACE2) receptor to facilitate this process [19]. ACE2 is widely expressed on arterial and venous endothelium, facilitating direct infection of endothelial cells by SARS-CoV-2 in multiple organs [20]. ACE2 converts angiotensin II (ATII) to angiotensin 1-7 [21]. Since SARS-CoV-2 utilizes ACE2 to enter human cells, ACE2 is internalized with the viral particles into endosomes, which may subsequently reduce ACE2 activity [22]. This downregulation of ACE2 expression upon viral entry results in increased levels of ATII and decreased levels of angiotensin 1-7 [23]. ATII exhibits proinflammatory and prothrombotic effects [24]. Reduced ACE2 activity in COVID-19 infection may lead to p38 upregulation [25]. In contrast, angiotensin 1-7 possesses anti-inflammatory and antithrombotic properties [25]. They bind Mas receptors on endothelial cells, thereby promoting vasodilation through enhanced production of nitric oxide and prostacyclin, which leads to the inhibition of platelet activation [26]. Consequently, the dysregulation of the RAAS in COVID-19 patients' vasculature triggers a sequence of events that increases coagulopathy. In addition to its direct effects on vasoconstriction, ATII has pro-oxidant properties and is a potent mediator of oxidative stress damage [27]. It interacts with the AT1R receptor to induce the rapid generation of reactive oxygen species (ROS) via NADPH oxidase (NOX) in endothelial cells, thereby triggering mitochondrial oxidative stress and ultimately leading to endothelial dysfunction [28]. As previously mentioned, angiotensin 1-7 induces the synthesis and release of nitric oxide from endothelial cells, playing a crucial antioxidant role and counteracting the effects of ATII [29]. Therefore, both the accumulation of ROS and the deficiency of nitric oxide lead to detrimental effects on the endothelium. The dysregulation of the RAAS induced by SARS-CoV-2 infection is illustrated in Figure 1.

There is a significant association between endothelial dysfunction and thrombotic events in various pathologies, including COVID-19 [30]. The glycocalyx in healthy endothelium plays a critical role in inhibiting clotting cascades, thus preventing microvascular thrombosis [31]. Degradation of the glycocalyx, observed in endothelial dysfunction, leads to the initiation of various clotting cascades [32]. For instance, endothelial damage or dysfunction contributes to thrombin generation and activation through the release of the procoagulant factor, FVIII [33]. Endothelial glycocalyx damage and degradation occur through the activation of sheddases, including heparanase, matrix metalloproteinases (MMPs), and hyaluronidase, by ROS and proinflammatory cytokines such as IL-1β, IL-6, and TNF-α [34]. MMPs play a critical role in glycocalyx damage by mediating the shedding of syndecan-1 and heparan sulfate [35]. Moreover, the degraded glycocalyx fragments further interact with endothelial cells, disrupting their barrier function [36]. Hyaluronic acid (HA) fragments or low molecular weight heparin (LMWH) has been reported to function as damage-associated molecular patterns (DAMPs), activating the innate immune response [37].

Furthermore, in COVID-19 infection, elevated vWF levels are closely associated with endothelial dysfunction and serve as a predictive marker for adverse outcomes [38]. vWF is a glycoprotein found in blood plasma, synthesized by endothelial cells and platelets [39]. It is stored in endothelial Weibel–Palade bodies and platelet α-granules and released following platelet activation [39, 40]. vWF serves two essential functions in hemostasis: It promotes the adhesion of platelets to subendothelial connective tissue and binds to blood clotting FVIII [41]. Virus entry contributes to inflammation and endothelial damage, where SARS-CoV-2-induced endotheliitis leads to the release of prothrombotic mediators, particularly vWF from Weibel–Palade storage bodies in endothelial cells, exposing underlying collagen to which vWF binds [42]. Elevated vWF levels indicate endothelial damage or dysfunction, enhancing platelet aggregation at the site of injury and excessive activation of the coagulation cascade, leading to thrombosis initiation [43].

In COVID-19, the immune response is dysregulated, primarily through alterations in three key components: the mitogen-activated protein kinase (MAPK) pathway, the complement system, and neutrophil function. First, MAPKs are considered central kinases that are activated early in the innate immune response and are divided into three subfamilies: ERK, p38, and c-Jun NH2-terminal kinase (JNK) [44]. SARS-CoV-2 infection promotes p38 MAPK activation [45]. MAPKs can be activated in COVID-19 disease due to dysregulated RAAS following ACE2 inhibition, as mentioned previously [25]. In addition, MAPKs are also activated by toll-like receptor (TLR) signaling and induce the expression of multiple genes that collectively regulate innate and inflammatory responses [46]. MAPK signaling plays an important role in innate immune responses by inducing the production of proinflammatory mediators such as cytokines (e.g., TNF) and chemokines [47]. The MAPK pathway mediates the potentiating effects of SARS-CoV-2 on platelet activation, thereby leading to thrombosis [48].

The complement system serves as a key component of innate immunity. Its activation leads to thrombosis by enhancing the coagulation cascade, inhibiting fibrinolysis, and activating both platelets and endothelial cells [49]. First, complement activation results in the upregulation of C5a, which subsequently increases tissue factor (TF) activity both in its circulating form and on endothelial cells [50, 51]. TF plays an important role in initiating the coagulation cascade via the extrinsic pathway (FVII) [52]. Furthermore, mannan-binding lectin serine proteases (MASPs) including MASP-1 and MASP-2 are key components of the lectin complement pathway and, in addition to this role, these serine proteases interfere with the coagulation and fibrinolytic cascades [53]. MASP-1 and MASP-2 directly cleave prothrombin, generating active thrombin [54]. Moreover, MASP-1 has thrombin-like activity; it independently activates fibrinogen and FXIII, thus promoting the crosslinking of fibrin and stabilizing blood clots [55]. Inhibitors of the complement system, such as C1 esterase inhibitor (C1-INH) and C4b-binding protein (C4BP), also exert inhibitory effects on the coagulation cascade [56]. C1-INH specifically inhibits FXII and thrombin, while C4BP inhibits protein S, which serves as a cofactor for the anticoagulant protein C [57–59]. Protein C, in turn, inactivates factors V and VIII [60]. Consequently, the activation of the complement system results in the activation of the thrombotic cascade. Fibrinolysis refers to the enzymatic process aimed at dissolving blood clots by breaking down fibrin [61]. In addition to its inhibitory effect on the coagulation cascade, C1-INH decreases fibrinolysis, by inhibiting plasmin [62]. Furthermore, complement factors induce the upregulation of plasminogen activator inhibitor-1 (PAI-1) in mast cells, thereby enhancing the inhibition of fibrinolysis and potentially promoting thrombosis [63, 64]. In vitro studies have demonstrated that MASP-1 can activate thrombin-activated fibrinolysis inhibitor (TAFI), an enzyme that downregulates fibrinolysis [55]. Therefore, fibrinolysis can be inhibited by both complement factors and complement inhibitors, by restricting the conversion of plasminogen to plasmin and inducing the expression of endogenous inhibitors of fibrinolysis. Additionally, complement factors C3 and the membrane attack complex (MAC) directly activate platelets and promote platelet aggregation [65]. Complement factors such as MAC can also induce endothelial activation, as it can trigger endothelial cells to release vWF [66]. The formation of the MAC on endothelial cell membranes appears to cause an influx of Ca^2+^ across the plasma membrane, resulting in an elevated cytosolic Ca^2+^ concentration, which leads to the secretion of VWF [67]. Furthermore, C5a stimulates the expression of P-selectin on endothelial cells, thereby promoting the recruitment and aggregation of platelets at sites of vascular injury [68, 69]. Therefore, activation of the complement system contributes to thrombosis by stimulating endothelial cells to secrete vWF and express P-selectin.

Neutrophils are an important component of the innate immune response [70]. In response to infection, neutrophils release neutrophil extracellular traps (NETs), which are composed of extracellular chromatin and microbicidal proteins [71]. These web-like structures not only hinder pathogen dissemination but also enhance the eradication of entrapped microorganisms [72]. However, excessive NET formation (NETosis) can be detrimental to the host and is implicated in various pathologies, including thrombosis [73]. Patients with severe COVID-19 disease exhibit elevated levels of NET components in their serum, including cell-free DNA, myeloperoxidase-DNA (MPO-DNA), and citrullinated histone H3 (Cit-H3) [74]. Hospitalized patients receiving mechanical ventilation show higher levels of DNA and MPO-DNA compared to those breathing room air [75]. The structural components of NETs are implicated as potent inducers of coagulation, capable of directly activating platelets and promoting thrombus formation. Specifically, cell-free DNA released by neutrophils triggers thrombin production in plasma by interacting with FXII [76, 77]. Furthermore, Cit-H3 molecules found on NETs activate platelets and enhance thrombin generation by interacting with platelet TLR2 and TLR4 [78]. Histones also stimulate platelets to release polyphosphates and expose phosphatidylserine and activated FV, thereby facilitating the assembly of the prothrombinase complex (FXa/FVa/Ca^2+^) [78]. Moreover, histones alter the activity of protein C, a natural anticoagulant, thus exacerbating their prothrombotic effects [79]. Neutrophil elastase and cathepsin G are additional components found in NETs that promote coagulation by degrading TF pathway inhibitors [80]. In NETs, the web-like structure of cell-free DNA enhances thrombus formation by acting as a scaffold for binding platelets, circulating erythrocytes, clotting factors such as TF, and procoagulant molecules including fibronectin and vWF [81–83].

### 3.2. Thromboembolic Events in COVID-19

Now that we have explored the theory around thromboembolic events and their onset in the physiological context of COVID-19, it is of interest to look at their actual incidence in practice. Various thromboembolic diseases in COVID-19 patients have been reported in the literature, including DVT, PE, MI, acute coronary syndrome (ACS), and acute limb ischemia (ALI). Such events have been reported in COVID-19 patients who were nonhospitalized, hospitalized, or even in the intensive care unit (ICU) [84, 85].

#### 3.2.1. Venous Thromboembolism (VTE)

Many diseases and illnesses are known to lead to an increase in thromboembolic events, especially those requiring hospitalization and placement in ICU [86, 87]. In fact, risk factors associated with severe illness and ICU placement favor VTE precipitation such as immobilization in hospital beds, old age, recent surgery, sepsis, and trauma, among others [86, 88]. In fact, a large retrospective study by Fang et al. on COVID-19 outpatients, with minimal risk factors associated with hospitalization, found that an increased risk of VTE in individuals infected with SARS-CoV-2 still exists [89]. A similar study done in the United Kingdom (UK) found that unvaccinated ambulatory COVID-19 patients had a hazard ratio (HR) for VTE within 30 days of infection of 21.42 when compared to noninfected controls [90].

With this newly arising association between COVID-19 and hematologic manifestations, the prevalence of VTE was studied over the years and followed in both COVID-19 patients and patients who recovered from it. We have chosen some meta-analyses/systematic reviews from each year within the 2020–2024 period and summarized their results in a table (Table 1). The overall prevalence of VTE in hospitalized acutely ill COVID-19 patients varies from one meta-analysis to another, and over the years, with an approximate range of 4.6% to 30% [87, 91, 92, 94]. Such a large interval is explained by the severity of the COVID-19 infection: Hospitalized patients have a greater prevalence of VTE [85, 94–96]. A nationwide study done in Sweden by Sjöland et al. demonstrated that their occurrence in hospitalized patients is significantly greater than in nonhospitalized patients [85]. By the same token, more severe infections and those requiring an ICU placement are stronger risk factors for VTE and arterial thromboembolism (ATE) compared to non-ICU cohorts [87, 91, 92, 95].

Furthermore, the risk of thromboembolic events is not restricted to the acute phase of COVID-19 infection, as it remains significant during the recovery period, with greater risk in the “early phase” of recovery [93]. A general trend seems to show that the more time passes after being infected with SARS-CoV-2, the less likely it is for an individual to develop a thromboembolic event [85, 93, 97], which further alludes to an interesting correlation with not only acute COVID-19 infection, but its sequelae and VTE events. For instance, the rate of VTE occurrence between 2 and 6 months after an infection is 16.91, compared to only 5.63 beyond 6 months (events per 1000 individuals) [85]. In the first meta-analysis investigating the occurrence of VTE events in patients recovered from COVID-19, significant HRs were calculated for both PE and DVT within 8.5 months of recovery compared to uninfected individuals (HRPE: 3.16 and HRDVT: 2.55, respectively) [93]. Nonetheless, the risk of thromboembolic events after hospitalization remains considerably lower than during hospitalization [85, 93].

PE and DVT are the main presentations of VTE events in COVID-19 infection and are distinguished by the lodging of a displaced thrombus at specific locations within the body. Porfidia et al. report in their paper that in infected patients, the emboli mainly involved the main trunk/lobar pulmonary arteries and segmental arteries of the lungs in 37.8% and 37.9% of PE cases, respectively, with the lowest risk of lodging in subsegmental arteries [91]. Meanwhile, DVT in COVID-19 disease, which presents as a precondition to PE, occurred twice as much within distal veins compared to proximal ones [91, 95]. An important consequence of VTE is an increased mortality risk [87, 88, 98]. In fact, Kollias et al. analyzed 17 studies in their paper comparing non-VTE COVID-19 to VTE COVID-19 patients and determined a statistically significant odds ratio for death in the latter group (OR: 2.1) [87]. An early retrospective study on patients in two hospitals in Wuhan further presents that 50% of the deceased patients from COVID-19 infection had some kind of coagulopathy, without further precision [99]. Despite such a strong association, there is not enough information in the literature to designate VTE as the direct cause of death in deceased patients infected with the virus [87]. However, in the autopsy of 12 infected patients, Wichmann et al. found that PE was the direct cause of death of 4 of them, even though more than half presented with a DVT [100].

The studies depicted in Table 1 demonstrate that the VTE prevalence varies much, ranging from 4.6% to 30% [87, 91, 92, 94]. This discrepancy can be addressed by the differing methodology of VTE diagnosis used in the existing retrospective cohort studies analyzed by the meta-analyses in the literature [87]. According to a letter by Shi et al., a closer look at the proportion of the COVID-19 patients undergoing diagnosis for PE/DVT in the published observational studies reveals an important inconsistency [101]. A possible surge from a pooled PE prevalence of 8% to 28% could be made when the entire hospitalized COVID-19 patient cohort was assessed for VTE, in contrast to only a fraction of the group [87, 101].

Multiple studies and meta-analyses propose certain risk factors for the increased occurrence of VTE events (Table 1). Recurrent associations with VTE which we already delved into are hospitalization, ICU placement, and severe illness in COVID-19 patients [87, 91, 92, 94]. Male sex [89, 91–94] and older age [89, 93] were found to significantly increase the risk of thrombotic events in COVID-19 infection, and this is expected, since both (especially the latter) are well-known risk factors for VTE regardless of an infection [102, 103]. Another interesting risk factor for blood clots in COVID-19 infection, which has also been studied in relation to VTE, is high levels of D-dimer [16, 87, 88, 92, 94]. The study by Suh et al. found that a D-dimer level superior to 500 μg had 96% sensitivity for the diagnosis of PE, whereas one greater than 1000 μg had 91% sensitivity with however low specificity for DVT [104]. During the pandemic, elevated D-dimer concentrations were therefore used as an initial marker for suspicion of DVT and therefore an indication for further diagnostic tests such as computed tomography (CT), pulmonary angiography, and other imaging tests, as per the recommendations of the European Society of Cardiology [87, 91, 105].

#### 3.2.2. ATE

Other thrombotic events were seen with an increased frequency during the COVID-19 pandemic, and they include ATE comprising MI, IS, systemic arterial thrombosis, and ALI [95, 106, 107]. In fact, the pooled frequency of ATE events in COVID-19 patients is reported in one meta-analysis by Candeloro et al. to be around 2%, varying from 0.4% to 9.6% [106]. A nationwide study on around 1.5 million COVID-19 patients in the United States of America (USA) also revealed an incidence of ATE of 3% [108]. Similarly to VTE, the risk of occurrence was greatest during the acute phase of infection [95], but some studies disagree on its significance after the acute phase: One study presents a HR for ATE events of 1.3 (95% CI: 1.2, 1.5) [109], whereas another finds a less significant risk with a HR of 0.7 (95% CI: 0.2, 2.2) [110]. Arterial thrombotic events also occurred in milder and even asymptomatic COVID-19 infections [107].

While an increase in mortality in COVID-19 patients could not with certainty be attributed to VTE, it would not be surprising to expect that MI or IS increases mortality risk. A case study by Khryshchanovich et al. on a COVID-19 patient with ALI of the iliofemoral segment demonstrates the importance of prompt diagnosis, as such a presentation is associated with a severe mortality risk [107]. Interestingly, although ATE events were associated with poor survival and a significant increased risk of death in patients hospitalized for COVID-19 by some [111, 112], it was not found to significantly increase mortality in COVID-19 patients in subsequent multivariable analyses, suggesting that ATE may not independently affect mortality outcomes [108]. This discrepancy between findings could be explained by a missed diagnosis of ATE in deceased COVID-19 patients, hence going back to the idea of requiring systematic autopsies. Nonetheless, in a retrospective cohort study by Gonzalez-Fajardo et al. where Kaplan–Meier survival curves for VTE and ATE were compared, it was found that ATE was more significant than VTE in predicting lower survival, especially if such events were recurring [113].

### 3.3. Bleeding Complications

A discussion about the thromboembolic events would not be complete without expanding on bleeding issues in COVID-19 patients. With thromboembolic events being looked at as the major hematologic risk in COVID-19, anticoagulant agents are provided for infected patients either at prophylactic or therapeutic doses [91, 114]. In a retrospective study, Lucijanic et al. suspect the use of such agents as being the cause of an increase in bleeding events in hospitalized COVID-19 patients [114]. Other causes can also be considered such as the host immune response discussed earlier [115]. In their study, 8% of the patients had undergone bleeding events, while only 3% had major bleeding events, in comparison with another study exclusively in COVID-19 ICU patients, reporting 23% of bleeding events, of which a quarter were major [114, 116]. Thus, the rate of bleeding events was significantly greater in severely ill COVID-19 patients and those admitted to the ICU, considering the dosage of anticoagulant provided for such patients [114–117]. Bleeding occurrences, especially major ones, were associated with increased mortality risk, where a study reports a HR of 1.55 in a COVID-19 ICU bleeding versus nonbleeding cohort [117]. Although such a number could allude to an important association, bleeding events are not considered as a “direct” cause of death in COVID-19 patients [114]. Recent clinical guidelines provide recommendations on the use of anticoagulation therapy in COVID-19 patients. These guidelines emphasize the importance of individualized treatment plans, considering both thrombotic and bleeding risks. For instance, the International Society on Thrombosis and Haemostasis (ISTH) recommends prophylactic doses of LMWH or unfractionated heparin (UFH) for all hospitalized COVID-19 patients, unless contraindicated due to bleeding risk [118]. In patients with a high risk of bleeding, dose adjustments or alternative therapies may be considered.

## 4. Discussion

COVID-19 infection has been shown to induce thrombosis through various mechanisms. First, SARS-CoV-2 infection dysregulates the RAAS [119]. Viral entry results in the downregulation of ACE2 expression, subsequently leading to increased levels of ATII and decreased levels of angiotensin 1-7 [120]. ATII exhibits proinflammatory, prothrombotic, and pro-oxidative effects [121]. Therefore, increased levels of ATII and decreased levels of angiotensin 1-7 result in the accumulation of ROS and a deficiency of nitric oxide, which adversely affects the endothelium [28, 29]. Endothelial damage or dysfunction promotes thrombin generation and activation through the release of the procoagulant FVIII [33]. SARS-CoV-2-induced endotheliitis leads to the release of prothrombotic mediators such as vWF [122]. Elevated vWF levels enhance platelet aggregation and excessive activation of the coagulation cascade, thereby triggering thrombosis. Moreover, SARS-CoV-2 infection results in dysregulation of the immune response, particularly affecting innate immunity through alterations in the MAPK pathway, the complement system, and neutrophil function [123]. Activation of the MAPK pathway triggers the production of proinflammatory mediators, including cytokines and chemokines [47]. This pathway also amplifies the effects of SARS-CoV-2 on platelet activation, contributing to the development of thrombosis [48]. Activation of the complement system enhances the coagulation cascade, inhibits fibrinolysis, and stimulates both platelets and endothelial cells, thereby contributing to thrombosis [49]. Complement factors and complement inhibitors both impede fibrinolysis by restricting the conversion of plasminogen to plasmin and inducing the expression of endogenous fibrinolysis inhibitors [124]. Additionally, complement factors C3 and the MAC directly activate platelets and facilitate platelet aggregation [125]. Complement activation also induces endothelial cells to secrete vWF and express P-selectin, further exacerbating the prothrombotic state observed in COVID-19 infection. SARS-CoV-2 infection leads to excessive formation of NETs, which can be harmful to the host and promote thrombosis [126]. The web-like structure of cell-free DNA in NETs acts as a scaffold that facilitates thrombus formation by binding platelets, erythrocytes, TF, fibronectin, and vWF [127]. The structural components of NETs directly activate platelets and enhance coagulation, thereby contributing to thrombosis.

Various thromboembolic events in COVID-19 patients have been reported in the literature, including DVT, PE, MI, ACS, and ALI. Such events have been reported in COVID-19 patients, whether in nonhospitalized, hospitalized, or even in ICU patients [84, 85]. The initial conjecture was that hospitalization, immobilization, trauma, and old age, which are essentially found in severe COVID-19 infections and in ICU patients, caused an increase in thromboembolic events [87]. However, the pathogenesis of COVID-19 discussed (dysregulated RAAS, endothelium, and immune response) was found to play an essential role in thrombosis in COVID-19 patients [87, 88, 91–94]. A herald study on this association is the multicenter cohort study led by Helms et al., which compared the occurrence of thromboembolic events in ICU patients with COVID-19-induced adult respiratory distress syndrome (ARDS) and non-COVID-19 ARDS [128]. With both groups treated with anticoagulants, there were significantly more elevated rates of thrombotic events in the COVID-19 ARDS cohort, with an odds ratio of 2.6 for such events, signifying COVID-19 ARDS patients are more than twice as likely to develop VTE than non-COVID-19 ARDS patients in the ICU [128]. Other studies also reinforced the idea that COVID-19 infection alone isolated from factors that accompany hospitalization can increase the occurrence of VTE [89, 90].

Most of the literature on VTE in COVID-19 infection converges on the idea that more severe COVID-19 infections that require hospitalization and ICU admission induce a greater risk for thrombosis [85, 87, 91, 92, 95]. In their meta-analysis, Starke et al. calculated a 27- and 7-fold increase in PE risk, respectively, in hospitalized COVID-19 patients and in the acute phase of the infection [95]. The risk also prevails post-COVID-19 infection during the recovery period, where the risk diminishes with time passed since the infection [85, 93, 97]. Massoud et al. concluded that a relevant risk for stroke remains in the first 7 days of recovery, which becomes insignificant only a week later [97].

Thromboembolic events in COVID-19 infections include VTE and ATE. VTE comprises PE and DVT, and their occurrence significantly reduces survival in COVID-19 patients [87]. With that said, it is hard to say with certainty that VTE is the direct cause of death in such patients, although many PE and/or DVT are found in deceased patients [87, 99, 100]. The need for more autopsies in deceased COVID-19 patients is indispensable to determine whether thromboembolic events are the direct cause of death in VTE-positive patients. Major risk factors for the development of DVT in COVID-19 patients seem to be hospitalization, ICU admission, male sex, aging, and elevated D-dimer levels [87, 89, 91–94, 104]. While male sex predisposes to an increased risk of VTE in the acute phase of COVID-19, in their systematic review, Zuin et al. established that within the first few months of recovery from COVID-19 infection, women were at greater risk of VTE [93]. Meanwhile, ATE comprising MI, IS, ALI, and other arterial systemic thrombotic events also occurs in COVID-19 patients with a lower frequency compared to VTE (around 2%–3%) [106, 108]. Studies in the literature however do not seem to find a common ground on whether ATE significantly increases the risk of mortality in COVID-19 patients [108, 111, 112].

### 4.1. VITT

Two types of vaccines have been developed against SARS-CoV-2: viral vector vaccines (specifically adenovirus-based vaccines) and mRNA vaccines [129]. Both types are designed to elicit an immune response, resulting in the production of neutralizing antibodies against the SARS-CoV-2 spike protein [130]. The mRNA vaccines, including Comirnaty (Pfizer) and mRNA-1273 (Moderna), consist of mRNA molecules encoding the SARS-CoV-2 spike proteins, which are encapsulated in lipid nanoparticles to facilitate efficient delivery into host cells [131]. In contrast, viral vector vaccines, such as ChAdOx1-nCoV (AstraZeneca), use modified chimpanzee adenoviruses that carry genetic material encoding the spike protein [132]. This genetic information is translated in the cytoplasm leading to the expression of the viral spike protein and initiation of an immune response [132].

VITT is an immunological reaction to adenoviral vector-based COVID-19 vaccines that leads to thromboembolic events following vaccine administration [133]. Overall, VITT is relatively uncommon and tends to occur in atypical sites such as the cerebral and splanchnic circulations [134]. The two-step mechanism underlying VITT is similar to the pathogenesis of heparin-induced thrombocytopenia, with both conditions caused by antiplatelet factor 4 (PF4) antibodies [135]. First, components of the adenovirus vector can combine with PF4, forming antigenic complexes [136]. Subsequently, this PF4/polyanion complex formation, along with the vaccine-induced proinflammatory environment, stimulates a B-cell response, leading to the production of anti-PF4/polyanion antibodies in VITT patients [137–139]. The binding of anti-PF4/polyanion antibodies to PF4 results in the formation of immune complexes that induce platelet and leukocyte activation, leading to the immunothrombosis observed in VITT [137, 140]. Specifically, these antibodies trigger platelet activation and aggregation by interacting with Fcγ receptor IIA on platelets [141]. Additionally, anti-PF4/polyanion antibodies stimulate neutrophils to release NETs in patients with VITT [142].

To face the increased risk of thrombotic events in COVID-19 infection, thromboprophylaxis is sought by physicians in the field. Administering prophylactic and therapeutic doses of anticoagulants to infected patients is highly recommended [91, 114]. The articles included in this review offer varying points of view with regard to the benefits of thromboprophylaxis in combatting thrombotic events. Porfidia et al. [91] and Agarwal et al. [92] assert in their meta-analyses that the risk of VTE remains significant despite the use of prophylactic anticoagulants (Table 1). On the other hand, Kollias et al. [87] found that in retrospective studies where anticoagulants were given to more than half of the cohort, there were significantly lower rates of VTE compared to studies where anticoagulants were used in less than 50% of the population [87] (Table 1). Another interesting finding was highlighted by Zuin et al. [93], who concluded that thromboprophylaxis does not lower mortality rate in infected outpatients, but does help in improving patient outcomes after hospitalization. Nonetheless, prophylactic anticoagulants are advised in severe cases of COVID-19 infection [95] regardless of the disagreements in the literature [91, 92]. In all cases, more large-scale studies need to be carried out to determine more precisely the usefulness of thromboprophylaxis.

### 4.2. The Pediatric Population, What Do We Know About It?

Until now, thromboembolism was not discussed in a specific age group, although it was mentioned that old age is an important risk factor for thrombosis in COVID-19 infection. However, in infected children, the risk of VTE or ATE still remains rare [143]. For instance, a large multicenter cohort study in the USA reported an incidence of 0.7% of such events in children asymptomatic for COVID-19, while it is 3 times greater in hospitalized children [144]. As in adults, the risk increases in children with other complications associated with COVID-19, such as severe COVID-19 infections, PICU admission, and especially multisystem inflammatory syndrome in children [143–145].

## 5. Conclusion

Thromboembolic events present a significant complication in COVID-19 patients, emphasizing the need for vigilant monitoring and prophylactic measures. The heightened risk underscores the interplay between viral infection and coagulation pathways, necessitating tailored anticoagulant therapies, as suggested by the guidelines. Further research is imperative to refine treatment protocols and improve patient outcomes. Addressing this complication holistically will enhance our overall management of COVID-19 infection. Additionally, comprehensive postrecovery care is crucial to mitigate long-term thromboembolic risks. Collaborative efforts in global research and clinical practice will be essential in developing effective strategies to combat these complications.

## Figures and Tables

**Table 1:** Characteristics and main thromboembolic findings in COVID-19 patients from systematic reviews and meta-analyses.

Reference	Authors	Country	Year	Number of studies	Total number of included patients	Prevalence			Main findings	Risk factors associated with VTE in COVID-19	Other findings
						VTE	DVT^∗^	PE^∗^			
[91]	Porfidia et al.	Italy	2020	30	3487	26%	14%	12%^a^	1. VTE incidence is greater in COVID-19 patients compared to sepsis/septic shock patients 2. VTE incidence is greater in ICU cohort of COVID-19 patients	ICU, male sex	i. Risk of VTE remained elevated despite the use of thromboprophylaxis ii. Main trunk/lobar and segmental arteries more commonly involved in PE iii. Distal veins more commonly involved in DVT

[87]	Kollias et al.	Greece	2021	47	6459	∼30%	27%	32%	1. Death odds ratio in COVID-19 patients with VTE is statistically significant compared to patients without VTE (95% CI: 1.2, 3.6) 2. Hospitalized patients with COVID-19 have a VTE prevalence of around 30%	ICU, elevated D-dimer levels	i. Studies where anticoagulants were given to more than 50% of the cohort have significantly lower rates of DVT

[92]	Agarwal et al.	United States	2022	28	6053	20.7%	—	—	1. VTE incidence is significantly greater in ICU cohort of COVID-19 patients and in those requiring mechanical ventilation 2. VTE events have no significant effect on increased mortality in COVID-19 patients	ICU, male sex, elevated D-dimer levels, low P/F ratio	i. Prophylactic anticoagulant did not significantly affect the prevalence of VTE ii. Patients with VTE had a longer length of stay at the hospital of around 4 days iii. Patients with VTE had significantly more elevated D-dimer levels

[93]	Zuin et al.	Italy	2023	6	2,060,496	—	2.3%^∗∗^	1.2%^∗∗^	1. Risk of VTE is greater in the acute phase compared to the recovery phase of COVID-19 disease 2. Women had a greater risk of VTE in the recovery phase, while having a lower risk during an acute infection	Acute phase of COVID-19, aging, male sex	i. Thromboprophylaxis does not help in reducing the mortality in COVID-19 patients but does help in recovering patients

[94]	Cénat et al.	Canada	2024	10	66,185	4.6%	—	—	1. Black ethnicity patients had greater VTE prevalence than other ethnic groups 2. Male patients had significantly greater VTE prevalence compared to female patients	Hypertension, male sex, hospitalization, elevated D-dimer levels, unvaccinated	i. Black individuals are more prone to develop VTE in the setting of COVID-19 due to: 1. Racial-associated increases in levels of D-dimer 2. Lower vaccination levels against COVID-19

Abbreviations: CI, confidence interval; DVT, deep vein thrombosis; ICU, intensive care unit; PE, pulmonary embolism; P/F ratio, PaO_2_/FiO_2_ ratio; VTE, venous thromboembolism.

^∗^Pooled estimates.

^∗∗^Prevalence in recovered COVID-19 patients (within 8.5 months of recovery).

^a^With or without DVT.

## Data Availability

Data sharing is not applicable to this article as no datasets were generated or analyzed during the current study.
